# Hemodynamic Response to the Head-Up Tilt Test in Patients With Syncope as a Predictor of the Test Outcome: A Meta-Analysis Approach

**DOI:** 10.3389/fphys.2019.00184

**Published:** 2019-03-07

**Authors:** Katarzyna Buszko, Sławomir Kujawski, Julia L. Newton, Paweł Zalewski

**Affiliations:** ^1^Department of Theoretical Foundations of Bio-Medical Science and Medical Informatics, Collegium Medicum, Nicolaus Copernicus University, Bydgoszcz, Poland; ^2^Division of Ergonomics and Exercise Physiology, Department of Hygiene, Epidemiology and Ergonomics, Collegium Medicum in Bydgoszcz, Nicolaus Copernicus University in Toruń, Toruń, Poland; ^3^The Medical School, Institute for Ageing and Health, Newcastle University, Newcastle-upon-Tyne, United Kingdom

**Keywords:** vasovagal syncope, head up tilt test, heart rate, stroke volume, blood pressure, meta-analysis

## Abstract

**Aim:** The paper presents a meta-analysis of studies comparing hemodynamic parameters: heart rate (HR), systolic blood pressure (sBP), diastolic blood pressure (dBP), and stroke volume (SV) measured during head-up tilt table test (HUTT) in patients with positive and negative HUT test outcome.

**Methods:** Pubmed and Clinical Key databases were searched for English-only articles presenting results of biosignals measurements during tilt test in patients suffering from syncope. From 3,289 articles 13 articles published between 1997 and 2015 investigating 892 patients (467 with positive HUTT outcome and 401 with negative one) were selected.

**Results:** There were not statistically significant differences observed between the parameters measured in supine position in patients with positive and negative test outcome [HR (*p* = 0.86), sBP (*p* = 0.32), dBP (*p* = 0.21), SV (*p* = 0.71)]. In tilt position the parameters HR and SV were significantly different when compared between the two groups of patients [HR (*p* = 0.02), sBP (*p* = 0.10), dBP (*p* = 0.59), SV (*p* = 0.0004)].

**Conclusions:** Changes in HR and SV parameters in response to tilt test turned out to be statistically significant. In supine position the differences between patients with positive and negative test outcome were not significant, hence tilt test can be considered as necessary in the diagnosis of vasovagal syndrome.

## Introduction

Syncope can be characterized as a temporary and self-finishing loss of consciousness. It usually results in a fall. It assumes that temporary, reversible global cerebral hypoperfusion (Parry et al., 2009) is the direct cause of a syncope. The syncope is often a cause of using the emergency services and for hospital admissions (Raj and Freeman, 2010). Each syncope episode should be an indication for a more in-depth diagnostic investigation because it can be indicative of a serious condition. In general, three types of syncope can be identified: cardiac, orthostatic and neuro-cardiogenic (Parry et al., 2009; Brignole et al., 2018). Our investigation is focused on the last of the mentioned types and it includes vasovagal syncope. This type of syncope can be triggered by the e.g., emotions, strong stress, a vein puncture or prolonged upright tilt, in particular in stuffy room (Parry et al., 2009). At present we cannot offer treatment of vasovagal syncope based on the elimination of its sources as its physiological mechanism is still not fully understood. Also, there is no diagnostic procedure for vasovagal syncope which would be commonly accepted as a gold standard. In general, a typical procedure for diagnosis of syncopal episodes is performed with Head Up Tilt Test (HUTT). In such test the patient is placed on a table that may be tilted to different angles (60–90° angle). Due to limited specificity and sensitivity (Forelo et al., 2012; Brignole et al., 2018) this test has not been accepted as the gold standard diagnostic procedure in diagnosing vasovagal syncope, however it has been in use since 1986 (Kenny et al., 1986). In fact, there is neither recognized alternative procedure to diagnose the syncope nor protocol of HUTT which would be recommended. The two most commonly used ones are the Westminster and the Italian protocols. In each protocol, the time in supine and tilt positions as well as the angle of the tilt is strictly defined. Nonetheless, it occurs that the tilt tests are performed upon the knowledge and experience of the clinicians. If the syncope does not occur in a passive-standing phase of the tilt test (lasting 45 min), then it is supported by pharmacological provocation [for example: nitroglycerine (0.4 mg sublingually), adenosine, isopropanol] combined with additional 20-min tilt. In the analyzed papers the result of the test was considered negative if syncope does not occur during that time. In fact, it does not mean that the patient does not have VVS. It only means that the patient has not been sufficiently provoked. In many studies the positive result of HUTT with or without provocation in the diagnostic procedure is treated generally as a positive result of HUTT without any special distinction between cases where provocation was or was not used.

During the HUTT typically the patient's electrocardiogram (ECG), blood pressure (BP) and sometimes impedance cardiogram (ICG) are recorded. During the syncope, the clinician categorizes the patient's response to orthostatic stress according to the VASIS classification (Vasovagal Syncope International Study by using analysis of recorded signals (ECG, blood pressure) (Brignole et al., 2018). There have been extensive attempts to describe the changes in monitored parameters observed in different time points between the tilt and the syncope. Until now, the researchers were focused mainly on identification of odds in the blood pressure, heart rhythm and impedance between patients with positive and negative results of HUTT test. Against this background, we considered that the prediction of the occurrence of the syncope without the necessity of a prolonged tilt but solely on the initial measurement made in a supine position or in response to orthostatic stress would be a mode useful diagnosis. This idea was the inspiration for our study. During the study we conducted a systematic review of research and performed meta-analysis to explore the possibility of HUTT test result prediction based on the parameters measured in the supine position and after tilt. We systematically reviewed the papers containing data from the tilt test measured in two groups of patients suffering from syncope (Catherine et al., 1997; Furlan et al., 1998; Shen et al., 2000; Fucà et al., 2006; Verheyden et al., 2008; Nigro et al., 2012; Tanriverdi Yilmaz et al., 2013; Koźluk et al., 2014; Freitas et al., 2015; Kim et al., 2015; Mitro et al., 2015; Russo et al., 2015; Sucu et al., 2015). The first group consisted of patients with the positive HUTT test result (HUTT(+)) and the second with the negative one (HUTT(–)). The details of our investigation are presented in the next sections.

## Materials and Methods

### Eligibility Criteria

A systematic review of published articles on the head-up tilt testing in patients with vasovagal syncope was performed. Review protocol was not published previously. Two investigators (SK and KB) independently searched Pubmed and Clinical Key databases in June 2018 for all full-text articles characterized by the subject “syncope AND tilt.” Duplicates were omitted during the analysis. The investigators analyzed all the titles to identify the potentially eligible articles, and they subsequently obtained the full texts to evaluate if they are eligible for the studies or not. Incompatible results were discussed with the third investigator (PZ). The criteria for results inclusion were as follows: English-only articles containing data before and immediately after head-up tilt testing with tilt angles varying between 60 and 90°, grouping patients based on positive (vasovagal) reaction on HUTT (HUTT(+)) and/or negative reaction (HUTT(–)) during studies. Only the papers with the full text access were qualified. The number of patients with positive and negative HUTT outcome ((HUTT(+)) and HUTT(–)) together with their mean age were presented in the Table 1.

**Table 1:** Main characteristics of the studies.

**References**	***N* (HUTT (+)) (mean age ± std)**	***N* (HUTT(–)) (mean age ± std)**	**Parameters**	**Methodology of HUTT**
Catherine et al., 1997	29 (6–16)	39 (6–16)	HR, sBP, dBP, SV	Unmedicated subjects remained in the 70° HUT for 6–7 min. Those who experienced near-fainting (defined as based on subjective symptoms or >20 mmHg sBP or >5 mmHg dBP) were tilted back as the first symptoms appeared. The protocol implies that the near-faint events were restricted to the last minute before tilt-back.
Furlan et al., 1998	22 (16 ± 1)	22 (20 ± 1)	HR, sBP, dBP	Unmedicated subjects remained in the 90° HUT for 15 min. The test was ceased whenever vasovagal episode criteria were fulfilled, such as subjective symptoms and increase in RR interval and a decrease of sBP for 80 and 40% comparing to the early tilt values, respectively.
Shen et al., 2000	16 (38 ± 15)	16 (39 ± 14)	HR, sBP, dBP, SV	Tilt in the 70° HUT lasted for 45 min. Each patient completed the sequential baseline tilt and then control group underwent isoproterenol administration. Vasovagal response was a criterion for test cessation.
Fucà et al., 2006	20 (40 ± 15)	20 (47 ± 11)	HR, sBP, dBP, SV	Unmedicated patients were tilted 60° HUT for 30 min or until syncope occurred. The test was ceased when syncope was induced. Spray nitroglycerin was administered sublingually (0.4 mg) and the test was prolonged for additional 15-min or until syncope occurred.
Verheyden et al., 2008	Drug-free 27 (32 ± 20)	NTG group 29 (41± 18)	HR, SV	Patients were assigned into two groups according to whether NTG was administered (0.4 mg sublingual after 20 min of 60° HUT) or not (according to Westminster protocol). Ten patients who developed pre-syncope during the HUT before NTG was administered were assigned into the group without drugs administration.
Nigro et al., 2012	I-mixed vasovagal syncope 60 (46 ± 22)	40 (36 ± 4)	HR, sBP, dBP, SV	HUT (60°) test was maintained up to 45 min or until syncope occurred. In case of a negative result of HUT, NTG was administered (0.4 mg sublingual after 20 min of HUT). The positive outcome of HUTT was defined in case of syncope co-occurring with hypotension and/or bradycardia. Classification was done according to a modified VASIS classification Kim et al., 2015.
Tanriverdi Yilmaz et al., 2013	97 (11.5 ± 2.9)	53 (11.7 ± 3.3)	HR, sBP	Passive phase with HUT (60°) was maintained for 45 min or until the syncope occurred. If no symptoms occurred during that time 0.4 mg of NTG was administered sublingually. The test was then prolonged for 15 min. Classification of syncope type was done according to a modified VASIS classification Kim et al., 2015.
Koźluk et al., 2014	45 (35 ± 20)	81 (36 ± 14)	SV	Passive HUT (60°) lasted for 45 min. In case of a negative result, 0.25 mg NTG was administered sublingually and the test continued for next 20 min. In case of contraindications to NTG, the test was continued without any drugs up to 60 min.
Mitro et al., 2015	28 (48.4 ± 17.1)	23 (44.6 ± 21)	HR, sBP, dBP, SV	HUT was performed according to the Italian protocol. HUT (60°) was maintained for 20 min or until occurrence of syncope. In case of no symptoms, sublingual nitroglycerine (0.4 mg in spray) was administered and the test was continued for another 15 min. The test result was defined as positive when patient developed syncope or presyncope associated with hypotension and/or bradycardia. Syncope was classified based on modified VASIS classification.
Russo et al., 2015	61 (37 ± 10)	34 (38 ± 11)	HR, SV	HUT (60°) was maintained for 20 min; in case of a negative result, pharmacological provocation with sublingual NTG (0.4 mg spray) was used. Tilting was maintained for another 15 min or until developing of syncope. The positive outcome of HUTT was defined in the case when syncope co-occurred with hypotension and/or bradycardia.
Sucu et al., 2015	33 (28.7 ± 11)	33 (30.8 ± 11)	HR	HUT (70°) lasted 20 min minimum. In case of negative result of a passive phase 0.4 mg of NTG was administered sublingually and the test was maintained until the syncope occurred or until 45 min of total protocol time. sBP ≤ 80 mm Hg and/or bradycardia or asystole co-occurrence with syncope or pre-syncope was defined as a positive test outcome.
Kim et al., 2015	28 (38 ± 15)	12 (43 ± 18)	HR, sBP, dBP	If the pre-syncopal symptoms did not occur during 20 min of HUT (70°), 0.25 mg of NTG was given sublingually and testing continued for another 20 min. Pre-syncopal symptoms were defined based on subjective symptoms, pallor, and sweating in association with hypotension and/or bradycardia. Syncope was prevented by means of tilt-back. Patients were assigned into two groups based on whether they experienced pre-syncopal symptoms after NTG administration or not.
Freitas et al., 2015	12 (32.3 ± 8.6)	12 (33.8 ± 5.9)	HR, sBP, SV	Unmedicated subjects remained in the 70° HUT during maximum of 40 min. The group consisted of patients without orthostatic hypotension, although experiencing frequent, typically repetitive neutrally mediated (NM) syncope episodes and with a positive HUT test outcome.

### Study Selection

**Figure 1:**
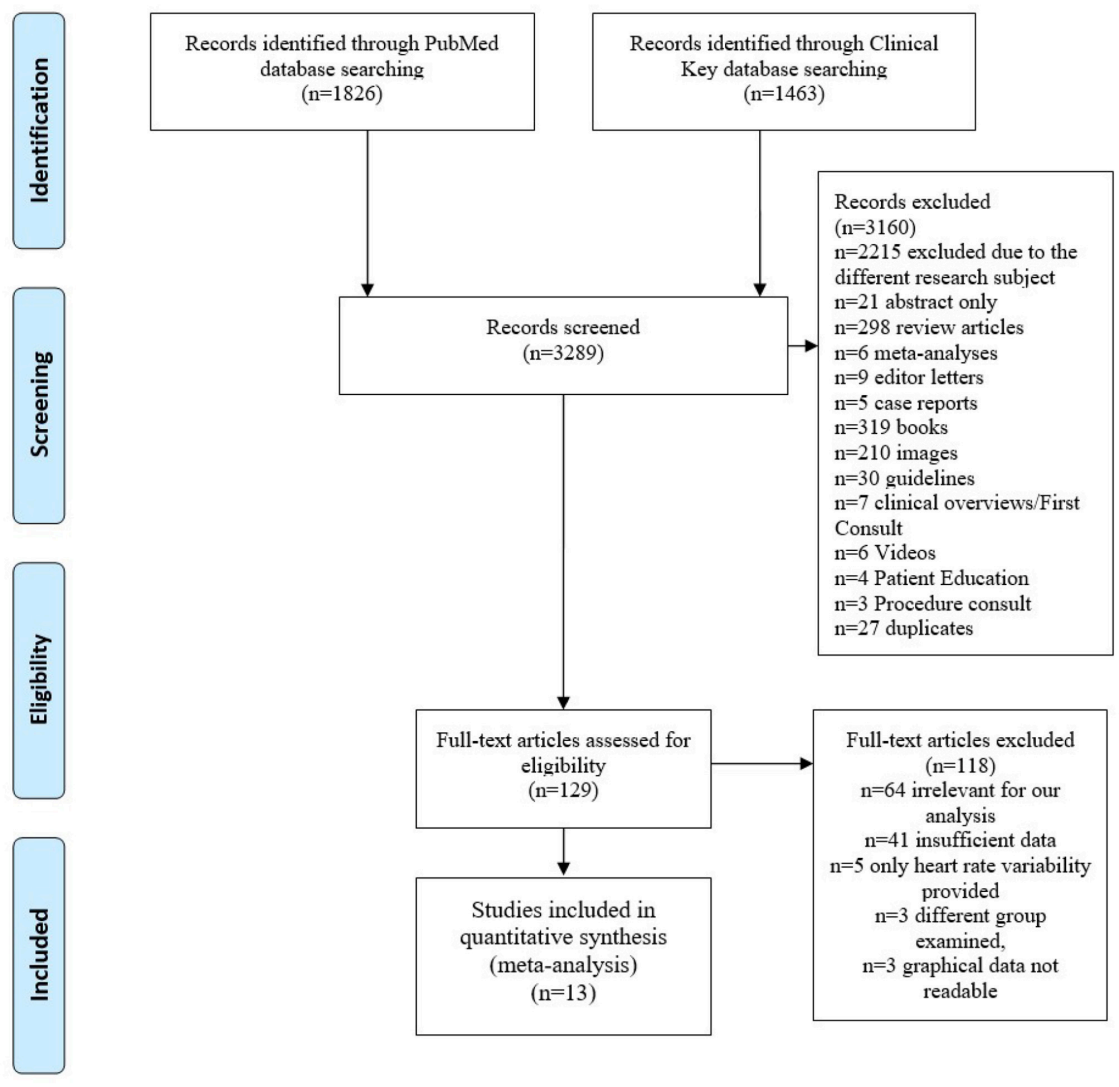
Flow diagram of the process of selection of study materials.

The investigators considered parameters of the tilt table testing protocols as follows: the presence and duration of the initial HUTT phase, the tilt angle. In Figure 1 we show the procedure of the study selection performed in the meta-analysis. Figure 1 illustrates study selection protocol. After entering the terms “syncope tilt” into the Clinical Key (full-text only) database, we obtained 1,463 full-texts. After entering “syncope tilt” into PubMed database (text availability: Full text) we obtained 1,826 journal articles. After an initial records screening, *n* = 3,160 records were excluded. *N* = 129 full-text articles were assessed for eligibility. We qualified only the papers with data: mean and standard deviation or standard error of the signals in supine position and tilt for two groups of patients (divided according to HUTT outcome). Finally, the results from *n* = 13 articles containing investigation of 892 subjects were included into meta-analysis. We tried to assess if there are differences between hemodynamics parameters in patients with positive and negative test outcome. We assumed the main research question as: could baseline measurements or the initial response on tilting serve as a predictor of syncope event.

Due to lack of agreed standards of diagnostics it was not possible to collect all the data from each paper (HR, sBP, dBP, SV). Therefore, different studies in the analysis for the separate signals were performed. The data for all the signals were included just in two papers (Shen et al., 2000) (Mitro et al., 2015). The data for HR, sBP and dBP were found in four papers (Furlan et al., 1998; Shen et al., 2000; Kim et al., 2015; Mitro et al., 2015). There were 6 papers that contained measurements of SV (Shen et al., 2000; Fucà et al., 2006; Nigro et al., 2012; Koźluk et al., 2014; Freitas et al., 2015; Mitro et al., 2015). Mean and standard deviation or standard error was extracted from each study. Extractions were duplicated, potential disagreements were discussed and resolved. Articles were screened for potential duplicates.

### Data Analysis and Statistical Methods

We noted measurements of the following biosignals recorded during head up tilt test: heart rate (HR), systolic blood pressure (sBP), diastolic blood pressure (dBP) and stroke volume (SV). We selected analyses with recordings performed in supine position and after the tilt. We performed a meta-analysis to compare two independent variables with the fixed effect model. The heterogeneity of the studies was analyzed by Cochran's Q statistics. The estimator T^2^ and I^2^ statistics ware also computed. All analyses were performed with a significance level α = 0.05. For each parameter the main results of the meta-analysis were showed as forest plots of mean value with 95% confidence intervals. We also performed publication bias assessment using Begg and Mazumdar test and Edgger test. The analysis was described visually by funnel plots. The analysis was performed with Matlab 2017b [MATLAB and Statistics Toolbox Release 2017, The MathWorks, Inc., Natick, Massachusetts, United States and Statistica 13.0 (StatSoft, Inc.)].

## Results

### Literature Search and Characteristics of the Studies

The main characteristics of the studies are shown in the Table 1.

### Comparisons Between Groups HUTT(+) and HUTT(–). A Meta-Analysis

**Figure 2:**
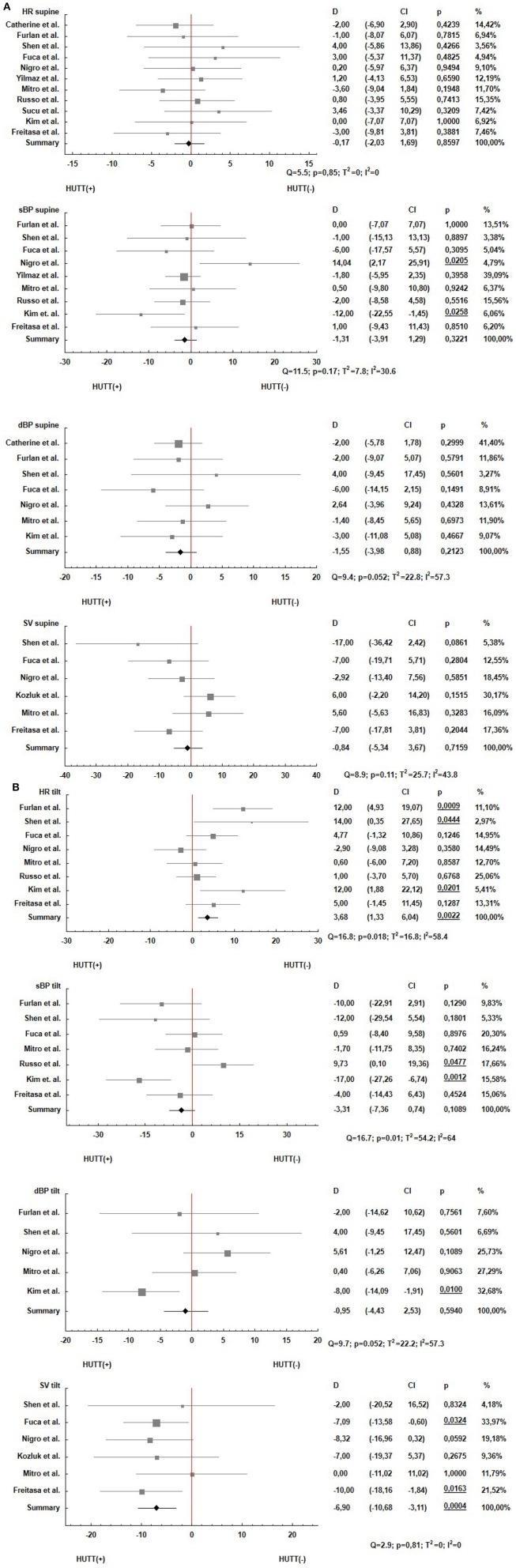
Forest plots estimating the parameters HR, sBP, dBP and SV for the patients with positive (HUTT(+)) and negative (HUTT(–)) tilt test outcome: **(A)** supine position, **(B)** tilt.

We summarized the results of the meta-analysis in forest plots. In Figure 2A results of the parameters comparisons in supine position are presented. Similar results for the tilt are presented in the Figure 2B.

In each figure the analysis of heterogeneity was included. On the top of each forest plot the Q statistics, *p*-value, T^2^ and I^2^ were shown. There are also included *p*-values for the summary results of the meta-analyses.

## Publication Bias Assessment

As we were aware that in our investigations based on meta-analysis we should pay special attention to the publication bias, we conducted the analysis for identifying and describing it. Therefore, we constructed funnel plots for each analyzed parameter, and we performed Egger test to examine the asymmetry of funnel plots. We also performed Begg and Mazdumar rank correlation test to examine the association between effect estimates and their variances. The results of the test are presented in the Table 2.

**Table 2:** The results of asymmetry assessment with Begg and Mazumdar test and Egger test.

**Parameter**	**Begg and Mazumdar test**		**Egger test**	
	**Coefficient**	***p***	**Coefficient**	***p***
HR-supine	0.23	0.31	1.4	0.21
sBP-supine	0.11	0.65	0.39	0.66
dBP-supine	0.05	0.88	0.4	0.62
SV-supine	−0.46	0.18	−4.3	0.07
HR-tilt	0.5	0.08	3.6	0.07
sBP-tilt	−0.24	0.45	−3.5	0.32
dBP-tilt	0.2	0.62	1.15	0.62
SV-tilt	0.6	0.09	1.2	0.27

**Figure 3:**
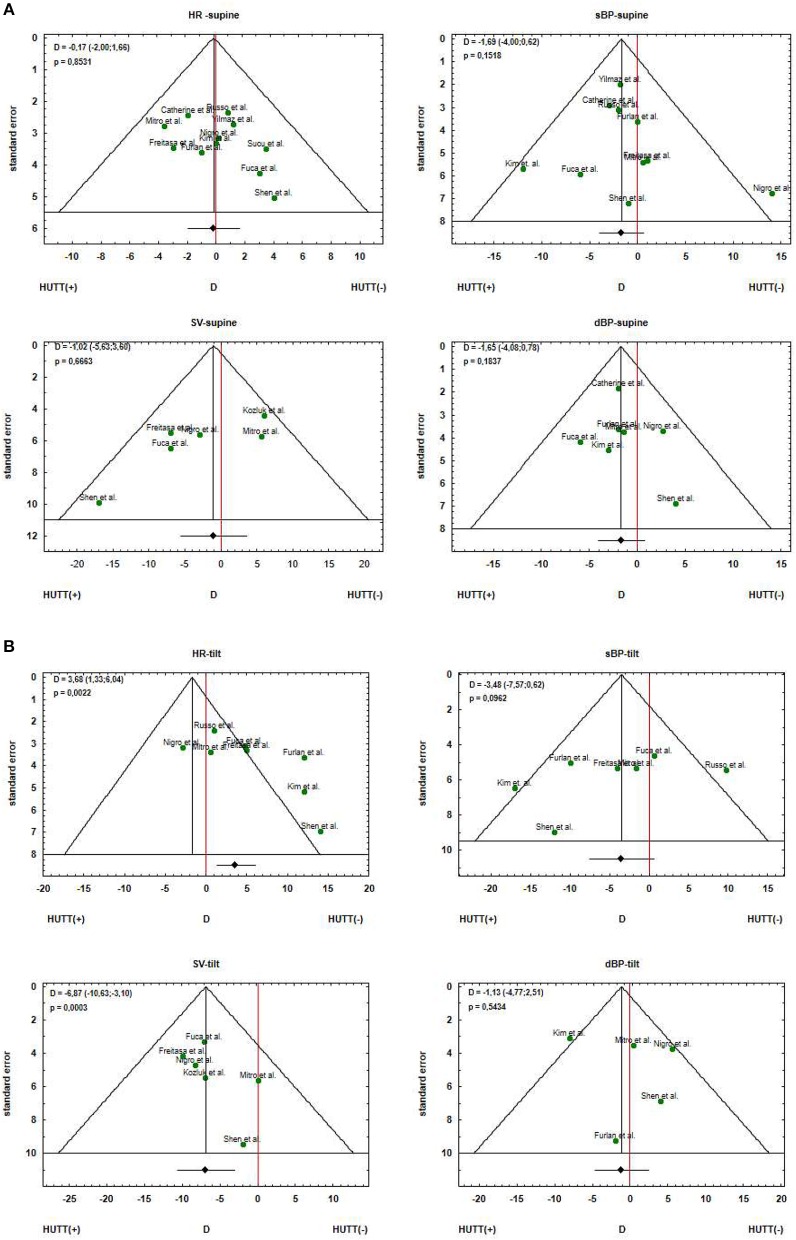
Funnel plots for the estimated parameters HR, sBP, dBP and SV for the patients with positive (HUTT(+)) and negative (HUTT(–)) tilt test outcome: **(A)** supine position, **(B)** tilt.

In the Figure 3 there are presented funnel plots for the parameters investigated in the meta-analysis. The plots in the Figure 3A correspond to analysis of measurements in supine position and Figure 3B corresponds to tilt position.

Funnel plot is informal and visual method of assessment of publication bias. This visual analysis suggests the asymmetry of the plot for SV in supine position and HR in tilt position. The skewed funnel plots may be caused by other factors than publication bias. The Begg and Muzdumar tests were not statistically significant and also the Egger tests were not significant for any of the parameters.

## Discussion

The meta-analysis was focused on the comparisons of hemodynamic parameters in patients with a positive and negative head up tilt test outcome. The comparisons allowed us to assess the possibility of HUTT outcome prediction based on the biosignals measurements in supine position and response to the tilt test. In the supine position we did not find any differences between patients with a positive and negative HUT test outcome (Figure 1A). Such result confirmed that the measurements of heart rate, blood pressure and stroke volume in supine position are insufficient in order to identify patients suffering from vasovagal syncope. Consequently the tilt test is necessary for such discrimination.

The main finding from our study is the statistically significant difference between the HUTT(+) and HUTT(–) group in HR and SV measurements in response to the tilt. This result indicates that prediction of the HUT test outcome is possible. One can expect that increased values of HR and decreased SV in response to tilt will result in positive HUTT outcome. The possibility of HUTT outcome prediction based on the heart rate (HR) measurement has been thoroughly investigated by Mallat et al. (1997). The authors proposed a criterion of negative tilt test result, which was based on the analysis of the heart rate (HR) in the first 6 min after an upright tilt. According to them, a slight rise of HR (≤18 bpm) in the first 6 min indicates a negative HUT test result. Ciliberti et al. (2018) compared HUT(+) patients with HUT(–). They found that very low frequency spectra component of heart rate during rest could be an independent predictor of syncope. On the other hand, Hear Rate Variability (HRV) and its dynamics during HUTT were not different in comparison between HUTT(+) and HUTT(–) groups of patients (Budrejko et al., 2018). Williams et al. (2018) showed the effectiveness of optimal control method as a predictor of time-varying quantities regulated by the cardiovascular control system. Moreover, they showed that pre-syncope was correlated with muscle oxygenation elevation, decreased skin blood flow and oxygenation (Lund et al., 2017). Considering practical implications Dorey et al. (2018) proposed using of knee-high compression socks in order to reduce the prevalence of syncope after physical exercise due to diminishing reduction in SV and thus cerebral blood flow velocity after moderate intensity. Ruzieh et al. (2018) showed that Closed Loop Stimulation pacing was effective in HUTT(+) patients being in the age of 40 years or older with cardio-inhibitory response. DePace et al. (2018) indicated the need for improvement of techniques of assessment of sympathetic and parasympathetic activation dynamics during HUTT to facilitate the subtype of syncope diagnosis.

In our research we found a significant difference in stroke volume in response to tilt (SV, *p* = 0.0004). Stroke volume (SV) is determined by ICG (impedance cardiography) measured continuously during the test. By using ICG signal it is possible to measure non-invasively the total electrical conductivity of the thorax as well as its changes in time. By using the impedance curve we may determine a time interval from opening to closing of the aortic valve (LVET) and stroke volume (SV). The obtained result shows that the impedance measurement during the tilt test should be included in the test along with the ECG and blood pressure (sBP, dBP) measurements. The inclusion of ICG measurement during the tilt test will allow researchers to predict test outcome based on SV analysis. Nonetheless, this interesting finding should be further confirmed by the research conducted with a sufficiently large group of patients whose diagnosis requires a tilt test which would need to be performed precisely in accordance with the defined protocol.

The HUT test has been used in diagnosis of unexplained syncope since 1986 (Kenny et al., 1986), but the gold standard in the test's protocols has not yet been set. The commonly used protocols are the Westminster and Italian ones (Brignole et al., 2018). However, tilt test methodology is still under discussion. The vital issue is primarily the tilt angle. In the first experiments 40° head up tilt angle was used (Kenny et al., 1986). The later studies performed by Fitzpatrick et al. showed that 60° angle in HUTT has high efficacy in detecting of vasovagal syncope (Fitzpatrick et al., 1991). Nowadays, the recommended angle of table in HUT test is between 60° and 70° due to experiments which showed high specificity associated with low sensitivity of the HUTT (Forelo et al., 2012). Therefore, in our meta-analysis we preferred the studies with table angle between 60° and 70° in tests protocols (Catherine et al., 1997; Shen et al., 2000; Fucà et al., 2006; Verheyden et al., 2008; Nigro et al., 2012; Tanriverdi Yilmaz et al., 2013; Koźluk et al., 2014; Kim et al., 2015; Mitro et al., 2015; Russo et al., 2015; Sucu et al., 2015). Just one paper presented results from the tilt test performed with 90° table angle (Furlan et al., 1998). Another critical point to discuss is that in some investigations the positive results of passive tilt test and the test with provocation were treated equivalently as a positive tilt test outcome. The data for those two protocols were not presented separately. However, the protocols with pharmacological provocation had higher sensitivity and lower specificity than passive tilt tests. Furthermore, the HUT test outcome depended on the type of pharmacological provocation. The commonly used provocative agents are nitroglycerin and isoproterenol. The comparisons of those two agents showed that the test stimulated with nitroglycerine had greater diagnostic capability (Forelo et al., 2012). In our research, we neither performed separate analysis for the passive and pharmacological protocols nor discriminated the test outcome on the basis of the provocative agents.

The observed heterogeneity of blood pressure (Figure 2B: sBP (*p* = 0.01, I^2^ = 64%); dBP (*p* = 0.052, I^2^ = 57.3%)] is a result of the described diversity of tilt test protocols used in many settings (Forelo et al., 2012).

The first conclusion is that in supine position the differences between patients with positive and negative test outcome were not significant. Due to this reason the prediction of HUT test outcome from baseline measurements of hemodynamics parameters (HR, sBP, dBP, and SV) is not possible. Hence, tilt test is necessary in the diagnosis of vasovagal syndrome. The second conclusion is that the obtained significant results for HR and SV in tilt position are of great importance for further research in the field of the tilt test outcome prediction. However, the critical points mentioned here in the head up tilt test methodology should be taken into consideration in further experiments. The third conclusion is that in the diagnosis of the vasovagal syndrome, the ICG should be included as a standard measurement in the head up tilt test.

### Limitations

In our meta-analysis we only considered articles published in English, hence we excluded results of the research published in non-English language texts. The substantial limitation is also above mentioned absence of gold standard of protocol in diagnosis procedure. We assume the negative result of HUTT as the result of the tilt test without syncope occurrence. In fact, the patients with negative HUTT result can suffer from syncope. The negative HUTT result does not necessarily mean not heaving VVS, but it may be a result of not sufficient provocation. In our research we did not consider aspects of age and gender. Furthermore, we did not perform separate analysis for different pharmacological agents.

#### Future Directions

The study performed on a sufficiently large group of patients with strictly defined HUT test protocol could confirm the results of this meta-analysis in reference to significant differences for HR and SV in supine position. Then the cut-off points for the parameters should be determined. Such experiment should also include age and gender.

## Author Contributions

KB: reviewed publications, analyzed the data, and wrote the paper; SK and PZ: reviewed publications; JN: manuscript preparation; All authors have read and approved the final version of the manuscript.

The study is funded by Collegium Medicum of Nicolaus Copernicus University and it did not receive any external funding.

### Conflict of Interest Statement

The authors declare that the research was conducted in the absence of any commercial or financial relationships that could be construed as a potential conflict of interest.
